# Layer-by-Layer assembled nano-drug delivery systems for cancer treatment

**DOI:** 10.1080/10717544.2021.1905748

**Published:** 2021-03-31

**Authors:** Xinyi Zhang, Tianying Liang, Qingming Ma

**Affiliations:** School of Pharmacy, Qingdao University, Qingdao, China

**Keywords:** Layer-by-layer assembly, nano-drug delivery systems, cancer therapy, chemotherapeutic drugs

## Abstract

Nano-drug delivery systems (NDDS) are functional drug-loaded nanocarriers widely applied in cancer therapy. Recently, layer-by-layer (LbL) assembled NDDS have been demonstrated as one of the most promising platforms in delivery of anticancer therapeutics. Here, a brief review of the LbL assembled NDDS for cancer treatment is presented. The fundamentals of the LbL assembled NDDS are first interpreted with an emphasis on the formation mechanisms. Afterwards, the tailored encapsulation of anticancer therapeutics in LbL assembled NDDS are summarized. The state-of-art targeted delivery of LbL assembled NDDS, with special attention to the elaborately control over the passive and active targeting delivery, are represented. Then the controlled release of LbL assembled NDDS with various stimulus responsiveness are systematically reviewed. Finally, conclusions and perspectives on further advancing the LbL assembled NDDS toward more powerful and versatile platforms for cancer therapy are discussed.

## 1. Introduction

Despite the enormous scientific efforts, cancer remains the most severe threat to humans and is currently the leading cause of death in patients globally (Mirzaei et al., 2016; Mattiuzzi & Lippi, 2020). When trying to achieve promising therapeutic effects toward cancer, one of the major challenges is the complicated microenvironment of the tumors, especially its complexity and heterogeneity (Sahu et al., 2018). Various chemotherapeutic drugs have been used to be circulated throughout the vast majority of tissues and organs in vivo, which is an effective way that achieve killing tumor cells (Cao et al., 2018). However, these drugs have inherent drawbacks, such as inadequate tumor tissues targeting, low biocompatibility and dose-limiting toxicity problems (Gidwani & Vyas, 2015). Therefore, novel methods for delivering chemotherapeutic drugs with higher therapeutic efficiency are highly desired.

**Figure 1.**
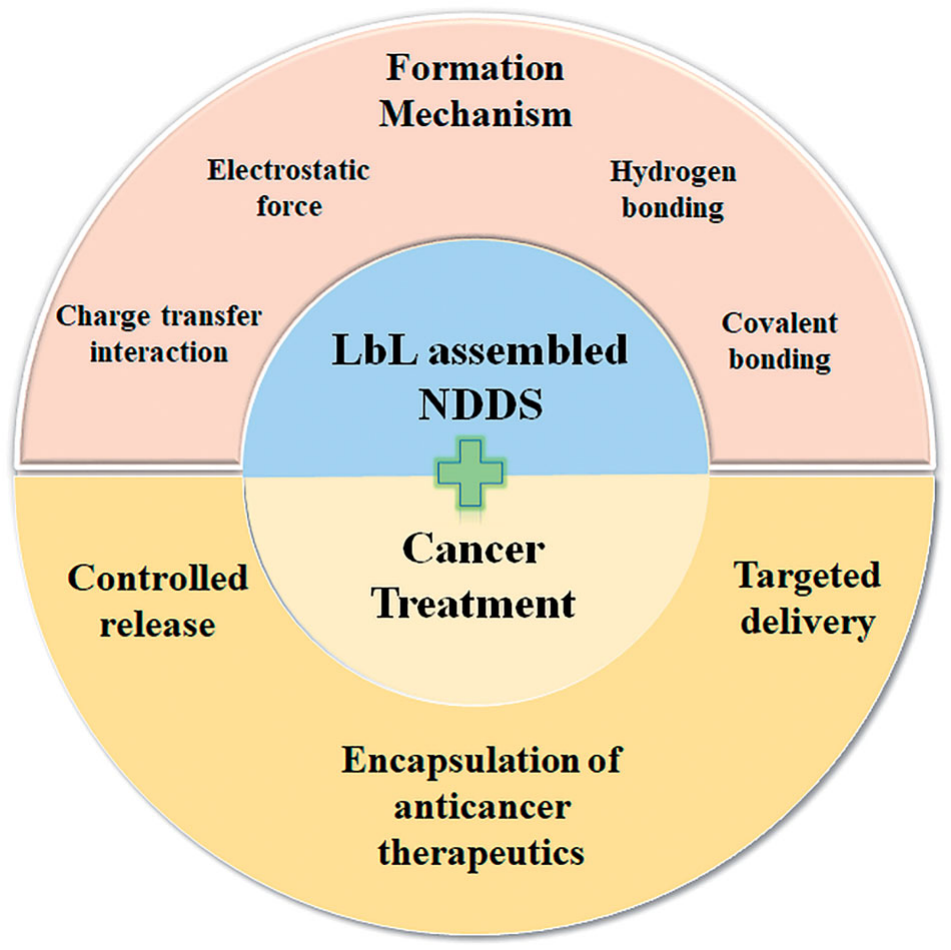
Overview of the layer-by-layer (LbL) assembled nano-drug delivery systems (NDDS) in cancer treatment including formation mechanism, and the encapsulation, targeted delivery and controlled release of anticancer therapeutics.

Nano-drug delivery systems (NDDS) are functional drug-loaded nanocarriers with diameters in the range of 10 to 1000 nm and composed of different natural or synthetic materials (Ma et al., 2020). Compared with conventional DDS at micrometer scales like microparticles, microcapsules and microspheres7, NDDS have attracted broad scientific interests for anticancer treatment in recent years due to distinct characteristics, including the capability of inducing EPR effect by the reduced size, the possibility of surface modifications for recognizing the attributes of the healthy cells and cancer cells in the clinical contexts, and concurrently target the cancerous tissues (Elias et al., 2010; Joshy et al., 2016; Joseph et al., 2017; Joshy et al., 2017; Bray et al., 2018; Joshy et al., 2018; Mohapatra et al., 2018; Dag et al., 2019; Thomas et al., 2019). One of the most promising methods that can generate such a kind of NDDS for cancer treatment is the layer-by-layer (LbL) assembly of multilayer films onto nano-templated nanoparticles, followed by optional template removal (Caruso et al., 1998; Tong et al., 2012; Ma et al., 2016; Park et al., 2018; Zhao et al., 2019). Compared with other techniques for generating NDDS, such as nanoprecipitation, solvent evaporation and in situ polymerization, the LbL assembly technology have its inherent advantages which make it more suitable to be applied in the fabrication of NDDS (Donath et al., 1998; Vautier et al., 2003; Deng et al., 2013; Gopi & Amalraj, 2016; Mohapatra et al., 2018; Dang & Guan, 2020). For example, compared with nanoprecipitation method which is typically used for the formation of nanoparticles by precipitation of a water insoluble polymer dissolved in a water miscible organic solvent upon addition to water, LbL assembly technology can not only achieve the formation of homogeneous nanoparticles, but can also generate other heterogeneous NDDS with different components and complicated structures like multilayers. Moreover, the thickness, surface charge and morphology of the multilayers can be well controlled by tuning the assembling conditions (Antipov & Sukhorukov, 2004). The fabrication process is simple with limited requirement of complicated conditions and equipment (Zhu et al., 2003). Besides, there is a strong chemical interaction between different layers, so that the LbL assembled NDDS have good thermal and mechanical stability (Ma et al., 2018). Further, both hydrophilic and hydrophobic drugs can be incorporated with optimized loading efficiency (Schneider et al., 2009). Overall, the unique functional and structural characters of LbL assembled NDDS makes it a great prospect in cancer treatment (Chen et al., 2017). Recently, plenty of researches and significant progress of LbL assembled NDDS for cancer treatment have been made. Therefore, a comprehensive depiction of the whole scene on LbL assembled NDDS for cancer treatment is desired. The contents of this review include: (1) the interpretations of the formation mechanism of the LbL assembled NDDS; (2) the tailored encapsulation of anticancer therapeutics in LbL assembled NDDS; (3) the elaborately targeted delivery of LbL assembled NDDS; and (4) the controlled release of LbL assembled NDDS with various stimulus responsiveness, as illustrated in Figure 1. We show an overview of LbL assembled NDDS for cancer treatment covering from fundamentals to progresses made for advanced anticancer therapeutic applications in recent years.

## 2. Formation mechanism of LbL assembled NDDS

The technique of LbL assembly is the consecutive deposition of complementary/interacting polymers onto colloidal particles, followed by optional removal of the sacrificial templates (Zhao et al., 2019). The technique first appeared in 1966 that method of alternate assembling of oppositely charged colloidal particles were used to prepare thin films (Iler, 1966). In 1990s, Decher et al. reported in detail for the first time that a multilayer film structure can be formed by alternately assembling oppositely charged polyelectrolytes on a flat plate through electrostatic interactions (Decher et al., 1994). Subsequently, Caruso et al. used removable colloidal particles as a template and assembled polyelectrolytes on the template through LbL technology (Caruso et al., 1998; Donath et al., 1998). The particles can be removed to obtain hollow capsules as vehicles for encapsulating various drugs. Ever since then, the technique of LbL assembly have attracted increasing interest, largely due to the ability to readily tailor their properties of the resultant nanocompositions, such as size, composition, porosity and surface functionality (Decher, 1997; Richardson et al., 2016; Guo et al., 2020). Moreover, the step-wise formation process of LbL assembly allows the introduction of multiple functionalities, thus providing opportunities to engineer a new class of nanoarchitectures with highly desired structures and functions (Ariga et al., 2014). The major driving forces for LbL assembly of NDDS include electrostatic force, hydrogen bonding, charge transfer interaction and covalent bonding. Recently, the works done by Prof. Soumitra Satapathi et al. and Prof, Veera Sadhu et al., especially those related with nanoparticles, are very helpful for illustrating the different types and wide applications of LbL assembled NDDS (Kim et al., 2015; Singh et al., 2017; Paliwal et al., 2019; Sur et al., 2019).

### 2.1. Electrostatic force

**Figure 2.**
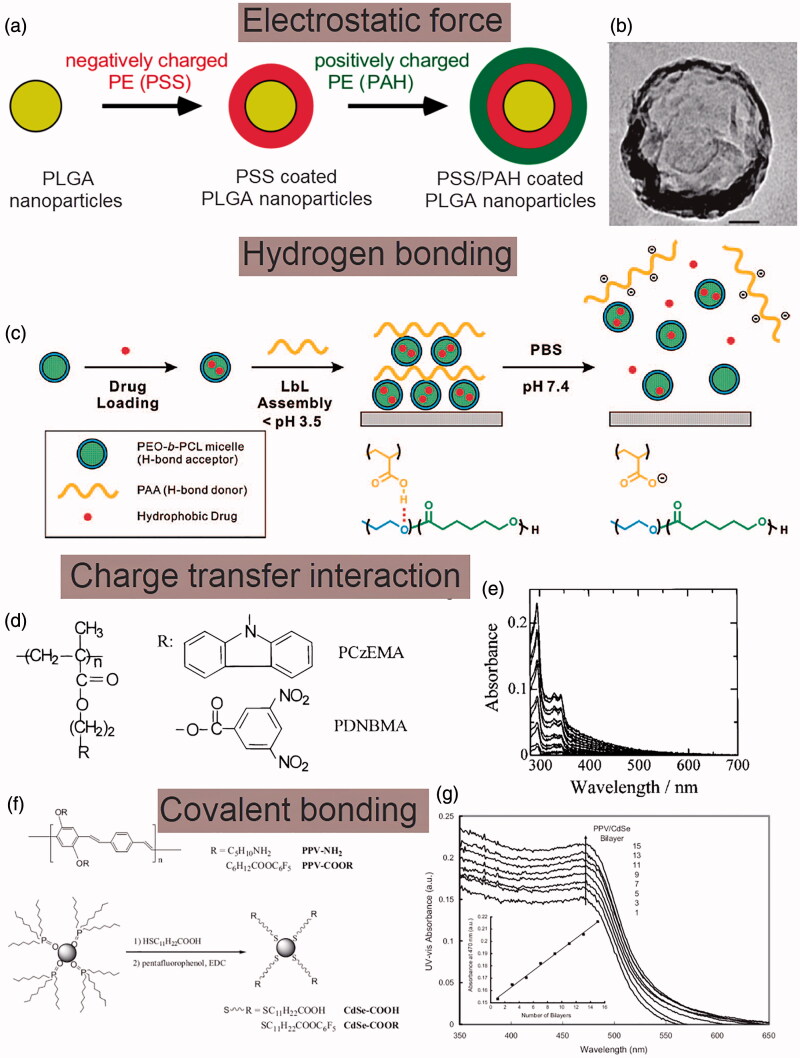
Formation mechanism of LbL assembled NDDS. (a) Schematic illustrations of the LbL assembly of PAH/PSS multilayers coated PLGA nanoparticles via electrostatic interactions. (b) Transmission electron microscopic (TEM) image of the assembled PAH/PSS multilayers coated PLGA nanoparticles, scale bar: 50 nm. (c) Schematics of hydrogen bonding LbL assembly of block copolymer micelles. (d) Chemical structures of PCzEMA and PDNBMA for charge transfer interaction based LbL assembly. (e) UV-vis absorption spectra of assembled PCzEMA/PDNBMA nanocompositions. (f) Chemical structures of PPV and CdSe for covalent bonding based LbL assembly. (g) UV-vis absorption spectra of the assembled PPV–CdSe nanocompositions. Inset shows the dependence of optical absorbance at 470 nm on the number of layers. (a, b) Reproduced with permission (Luo et al., 2012). Copyright 2012, Wiley-VCH. (c) Reproduced with permission (Kim et al., 2008). Copyright 2018, American Chemical Society. (d, e) Reproduced with permission (Shimazaki et al., 1998). Copyright 2008, American Chemical Society. (f, g) Reproduced with permission (Liang et al., 2006). Copyright 2018, Wiley-VCH.

The electrostatic interactions between oppositely charged ions are the most applied driving force in the LbL assembly of NDDS. The principle is to alternately deposit different oppositely charged materials like polyelectrolytes on a selected nano-template, and then remove the template to obtain multilayer composite films with the structure and thickness accurately controlled (Duan et al., 2018). For instance, by using the electrostatic interactions between negatively charged polystyrene sulfonate (PSS) and positively charged polyalylamine hydrochloride (PAH), bi-layers of PAH/PSS can be formed on the surface of PLGA nanoparticles, as illustrated in Figure 2(a) (Luo et al., 2012). Moreover, due to the nonspecific electrostatic interactions, functional components, such as conductive polymers, photopolymers and biological macromolecules, can be easily incorporated into the films to form functionalized composite membrane (Liu et al., 2019). For example, hemoglobin can be assembled with PSS to form hemoglobin/PSS coated nanoparticles while keeping the biological activity of hemoglobin intact (Liu et al., 2004).

### 2.2. Hydrogen bonding

Hydrogen bonding is a force slightly weaker than electrostatic interaction. Unlike electrostatic forces-driven LbL assembly which requires the film-forming materials capable of being charged, hydrogen bonding can also be applied to broaden the types of materials eligible for LbL assembly (Sham & Notley, 2015; Hwangbo et al., 2016). For instance, hydrogen bonding between poly(acrylic acid) (PAA) as an H-bond donor and biodegradable poly(ethylene oxide)-block-poly(caprolactone) (PEO-b-PCL) micelles as the H-bond acceptor have been applied to facilitate the assembly of nano-sized vehicles composed of PEO-b-PCL/PAA under acidic conditions, as illustrated in Figure 2(c). The proposed hydrogen bonding based LbL assembly of nano-sized vehicles can provide a general means to deliver anticancer therapeutics with controllable release properties (Kim et al., 2008). It is worth noting that compared with electrostatic force, hydrogen bonding is more sensitive to the change of environmental factors, such as pH value, temperature and ionic strength, resulting in the prepared NDDS unstable and easy to dissociate under the change of the microenvironment (Sukhishvili & Granick, 2002).

### 2.3. Charge transfer interaction

Charge transfer interaction is the major driving force for the LbL assembly of two nonionic polymers. The resultant nanocompositions exhibit various unique physical and chemical properties, such as electrical conductivity, neutral-ion phase transition, nonlinear optical properties, and good hydrophobicity (Meier, 2005). For instance, Shimazaki *et al* reported the use of charge transfer interactions for LbL assembly by demonstrating the assembly between electron-donating carbazolyl groups and electron-accepting 3, 5-dinitrobenzoyl groups in the side chains of two kinds of methacrylate polymers, poly [2-(9-carbazolyl) ethyl methacrylate] (PCzEMA) and poly [2-[(3, 5-dinitrobenzoyl) oxy] ethyl methacrylate] (PDNBMA), respectively, as shown in Figure 2(d,e) (Shimazaki et al., 1998).

### 2.4. Covalent bonding

The stability of NDDS assembled by electrostatic interaction, hydrogen bonding and charge transfer interaction are relatively poor and susceptible to corrosion in polar solvents and high-concentrated salt solutions (Zhang et al., 2021). Recently, covalent bonding-based LbL assembly has been proposed. The energy of covalent bonding is higher than that of the electrostatic force and hydrogen bonding, therefore the prepared NDDS have better stability (Bergbreiter & Liao, 2009). Moreover, the advantage of covalent bonding-based LbL assembly is that the preparation process can be extended from the aqueous system to organic solution system, therefore uncharged and water-insoluble functional polymers can be used for the assembly of NDDS (An et al., 2018). For instance, the covalent bonding between CdSe nanoparticles and p-conjugated polymers poly(p-phenylenevinylene)s (PPVs) have been applied to facilitate the LbL assembly of nanocompositions, as illustrated in Figure 2(f,g) (Liang et al., 2006).

### 2.5. Long-term stability of LbL assembled NDDS under physiological conditions

The long-term stability of LbL assembled NDDS is one of the major concerns *in vivo*, particularly under physiological conditions since most of these multilayers will be disassembled under physiological conditions (Tong et al., 2005; Jia et al., 2011). Cross-linking is an effective way to enhance the long-term stability and tune the properties of the nano-multilayers to survive through harsh physiological conditions including high ionic strength, extreme pH and strong polar organic solvent (Wang et al., 2007). Moreover, cross-linking can effectively manipulate the permeability and mechanical strength of LbL assembled NDDS. One of the representative crosslinking methods is carbodiimide chemistry. Uncross-linked components can be selectively released at higher pH, yielding single component and hydrogel-like NDDS. These NDDS exhibit reversible pH-responsive swelling and shrinking, which can be used for loading and releasing anti-cancer therapeutics (Liu et al., 2019).

## 3. Encapsulation of anticancer therapeutics in LbL assembled NDDS

It is well recognized that there are various factors hinder the efficient encapsulation and delivery of anticancer therapeutics in cancer treatment, such as poor permeability in solid tumor tissues, difficulty for nano-drug carriers to reach the deep tumor, and strong side-effects caused by the systemic distribution of drugs. By applying the LbL assembled NDDS, more efficient drug encapsulation can be achieved (Ai, 2011; Liu & Picart, 2016; Cheng et al., 2016; Cheng et al., 2017; Li et al., 2020).

### 3.1. Encapsulation of anticancer therapeutics

**Figure 3.**
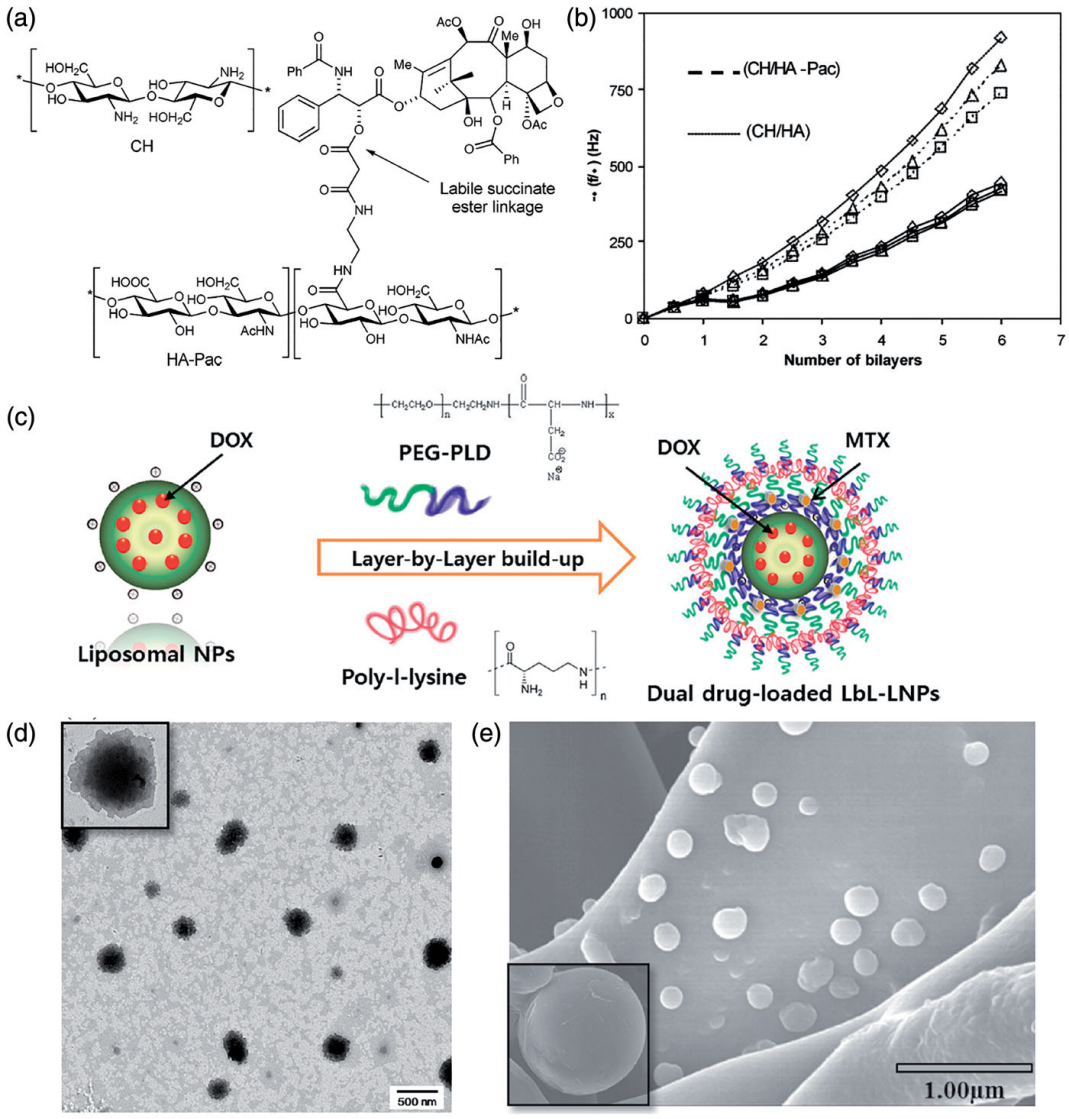
(a) Structure of the HA prodrug of paclitaxel (HA-Pac) and CH. (b) Quartz crystal microbalance (QCM) resonance frequency shifts as a function of the number of layers with different harmonics. (c) Schematic illustration of the fabrication of LbL-coated LNPs. (d, e) TEM and SEM images of the assembled LNPs. (a, b) Reproduced with permission (Thierry et al., 2005). Copyright 2015, American Chemical Society. (c, d, e) Reproduced with permission (Ramasamy et al., 2014). Copyright 2014, Elsevier.

Biological macromolecules, such as peptides, proteins, nucleic acids and most polysaccharide molecules, have been widely used as anticancer therapeutics. These macromolecules can be well dissolved or dispersed in aqueous solutions and carry a large number of charges by which can be easily encapsulated in the LbL assembled NDDS by electrostatic interaction (Ma et al., 2020). This principle can be further extended to all charged water-soluble macromolecular drugs and hydrophilic small molecule drugs. While the uncharged hydrophobic drugs cannot be directly incorporated on the NDDS like hydrophilic drugs, various methods have been proposed to achieve the successful encapsulation (Schneider et al., 2009). A straightforward approach is to directly incorporate the hydrophobic drugs into the hydrophobic inner cavity of NDDS, such as oil nanodroplet, mesoporous silica nanoparticles, micelles and liposomes. Besides, covalently bonding is also adopted to assemble the drug with feasible electrolyte molecules to form macromolecular prodrugs that can be subsequently assembled into NDDS. For instance, the anticancer drug Paclitaxel (Pac) can be assembled with hyaluronic acid (HA) through self-cleavable ester bonds to form prodrugs, then the obtained prodrugs can be further assembled with chitosan (CH) by LbL technique to form multilayered Pac-HA/CH NDDS, as shown in Figure 3(a,b) (Thierry et al., 2005). When subjected to physiological conditions, the generated NDDS can achieve sustained release of Pac by breaking the ester bond.

### 3.2. Co-encapsulation of two chemotherapeutic drugs

LbL liposomal nanoparticles as deliver vehicles for co-delivery of doxorubicin (DOX) and mitoxantrone (MTX) simultaneously has been developed, as illustrated in Figure 3(c) (Ramasamy et al., 2014). Specifically, LbL assembly can be achieved by sequential deposition of poly-L-lysine (PLL) and poly(ethylene glycol)-block-poly(L-aspartic acid) (PEG-b-PLD) on liposomal nanoparticles (LbL-LNPs), which subsequently generates spherical and stable multilayered NPs with 240 nm in size, enabling effective systemic administration. The functional groups and compartments in the shell and core facilitate the loading of DOX and MTX. The pharmacokinetic results of male rats show that the LbL-LNPs can significantly reduce the clearance rate of the two drugs and prolong their circulation time in the body.

### 3.3. Co-encapsulation of two gene therapeutics

LbL assembled NDDS can facilitate the co-delivery of gene therapeutics. For instance, starting with gold nanoparticles as a core, LbL degradable polymer coatings enable the simultaneous co-delivery of DNA and small interfering RNA (siRNA) (Bishop et al., 2015). In culture of human breast tumor cells labeled with green fluorescent protein gene, the delivered DNA can be smoothly expressed in the cells, and the gene silencing effect of siRNA is better than that of commercially available transfection reagents. Further, the same method can be used to co-deliver two kinds of plasmid DNA, and by tuning the assembly order, different DNA expression time can be achieved (Bishop et al., 2016).

### 3.4. Co-encapsulation of chemotherapeutic drug and gene

Combination of chemotherapeutic drug and siRNA can affect multiple disease pathways and show great promises in suppressing tumor progression, therefore co-delivery of drug and gene within a same NDDS offers a vital means in cancer therapy (Li et al., 2013; Tsouris et al., 2014). A pH-sensitive liposome to co-deliver drug and siRNA to tumor region has been developed. Driven by the electrostatic interaction, the pH-sensitive material, carboxymethyl chitosan (CMCS) has been coated onto the surface of the cationic liposome (CL) to form the CMCS-modified pH-sensitive Sf/siRNA co-delivery cationic liposome (CMCS-SiSf-CL) (Yao et al., 2015). The chemotherapeutic drug Sorafenib (Sf) can be loaded in the cationic liposome core, and siRNA can be incorporated loaded on the liposome multilayer. The release rate of Sf and siRNA from the resultant CMCS-SiSf-CL exhibit pH-sensitive release behavior.

### 3.5. Encapsulation of protein and peptide drugs as building blocks in the LbL assembled NDDS

Since most of the biologically active macromolecular drugs, such as nucleic acids, proteins, and peptides, have a large amount of surface charges themselves, these drugs can be directly loaded into the LbL assembled nanolayers as assembly components. For instance, Lvov et al. first reported the concept of layer-by-layer assembly of DNA and synthetic polycation polyacrylamide (Hou et al., 2002). DNA can be released from the layers in a controlled manner; apart from DNA, other peptides like functional plasmid DNA or oligonucleotides have also been successfully encapsulated in the layers as building blocks. Moreover, other component materials such as polyacrylamide and polyethyleneimine that can interact with DNA has also been incorporated in the layers to offer the formed NDDS special properties like biodegradability and certain response characteristics (Shi et al., 2002; Hu et al., 2008; Lu et al., 2008; Jiang et al., 2014; Shang-Tse et al., 2015). Further, enzyme-sensitive polyelectrolytes and DNA have been used to assemble an enzyme biodegradable gene delivery system, which demonstrated a way to degrade controlled-release DNA by multilayer membranes only in the presence of specific enzymes (Ren et al., 2006). Although the research on gene multilayer film mainly focuses on DNA, there are also reports on RNA functionalized multilayer film (Freund et al., 2012). Some growth factors such as BMP, VEGF and hydrophilic functional short peptides can also be incorporated into multilayer membranes by this method (Etienne et al., 2006; van den Beucken et al., 2007; Belda Marín et al., 2020; Rangel et al., 2021).

## 4. Targeted delivery of LbL assembled NDDS

Achieving the controlled release of the delivered anticancer therapeutics at the targeted tumor location is the one of the major strategies in chemotherapy (Li et al., 2020). The goal of targeted delivery is to obtain high enough local concentrations of drugs together with low systemic exposures. The tunable nanocompositions of LbL assembled NDDS can provide offer versatile binding groups for connecting different targeting ligand and therefore facilitate the targeted delivery of the encapsulated anticancer therapeutics. Various scholars have recently reported progress and achievements in the targeted delivery of LbL assembled NDDS, including Prof. Behrooz johari et al., Prof. Fatemeh Salahpour Anarjan et al. and Prof. Fatemeh Salahpour Anarjan et al. who have systematically explain the delivery mechanism of anticancer drugs by LbL assembled NDDS to achieve superior therapeutic effects (Cui et al., 2016; Salahpouranarjan, 2019; Gharbavi et al., 2020a; 2020b; Salahpour-Anarjan et al., 2021). Moreover, Polydopamine (PDA), possesses many properties, such as a simple preparation process, strong adhesive property, outstanding photothermal conversion efficiency, easy functionalization and conjugation targeting ligand. PDA has attracted increasingly considerable attention because it provides a simple and versatile approach to functionalize material surfaces for obtaining the LbL assembled NDDS (Zhu et al., 2016; Cheng et al., 2017; Peng et al., 2018; Wang et al., 2020; Zeng et al., 2020).

### 4.1. Passive targeting

Passive targeting refers to the delivery of drug to specific organs through spontaneous physiological processes. The phenomenon of ‘enhanced permeability and retention effect (EPR)’ has now became the gold-standard in passive targeting of anticancer drugs, which is applicable for almost all rapidly growing solid tumors (Bazak et al., 2014; Kang et al., 2020). LbL assembled NDDS in the range of 5 0 ∼ 200 nm tends to accumulate in tumor tissues spontaneously due to EPR effect, facilitating the achievement of passive targeted delivery of anticancer therapeutics with enhanced therapeutic efficiency (Hirsjarvi et al., 2011; Szczepanowicz et al., 2016).

**Figure 4.**
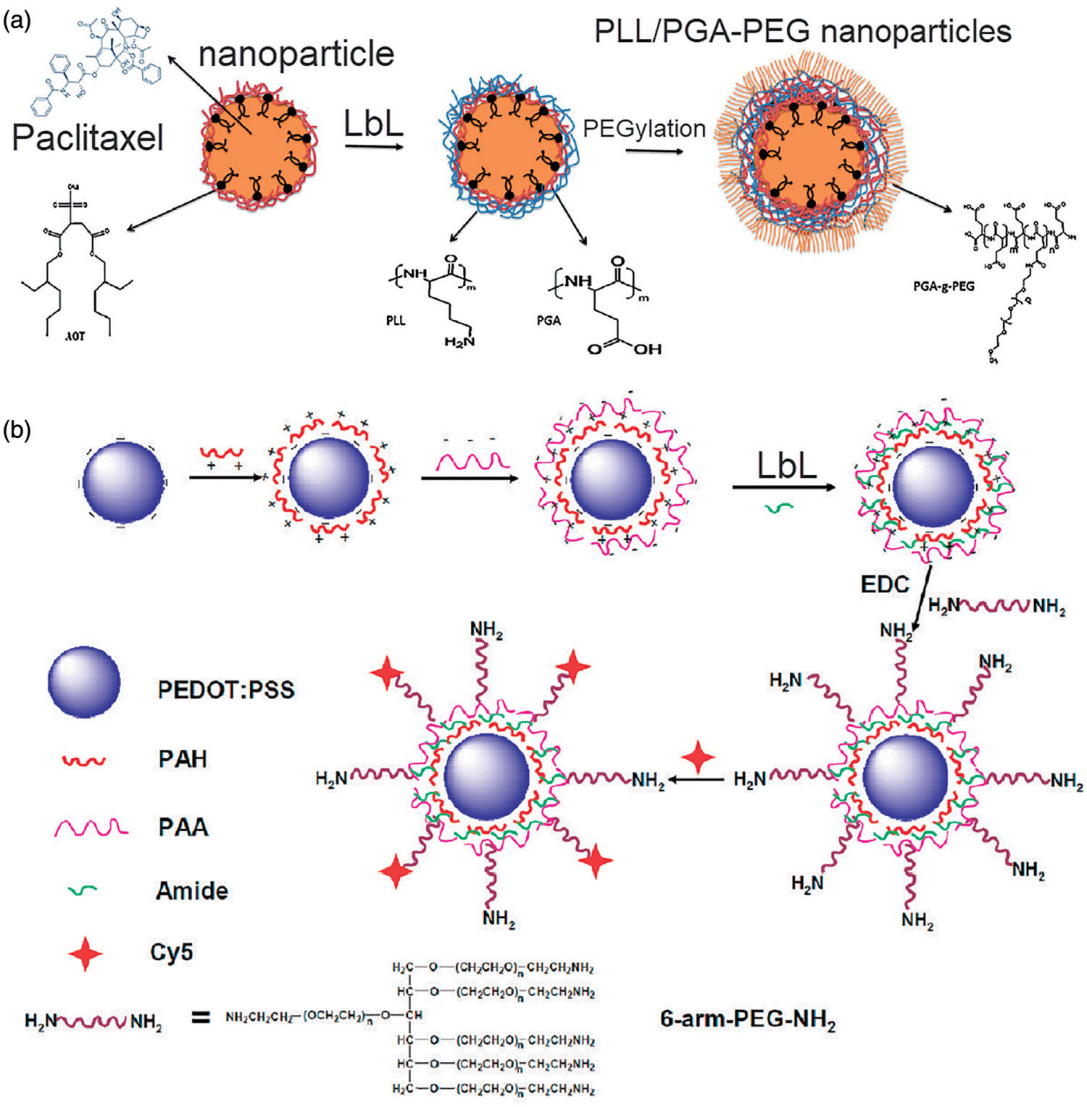
Passive targeting delivery of LbL assembled NDDS. (a) Schematics of the LbL assembly of the PEGylated PLL/PGA nanoparticles for the passive and prolonged delivery of paclitaxel. (b) Illustrations of the LbL assembly of the PEDOT/PSS-PEG nanoparticles. (a) Reproduced with permission (Szczepanowicz et al., 2016). Copyright 2016, Elsevier. (b) Reproduced with permission (Cheng et al., 2012). Copyright 2018, American Chemical Society.

Moreover, in the physiological environment, LbL assembled NDDS are inclined to be eliminated by the phagocytes in the reticuloendothelial system (RES) of the body and eventual degradation of the NDDS or early release are the basic factors that limit deliver efficiency (Hirsjarvi et al., 2011). Therefore, a prerequisite for efficient drug delivery is that the LbL assembled NDDS with the encapsulated anticancer therapeutics can circulate long time enough in the bloodstream. Further, prolonged circulation time also enables passive targeting as the NDDS can pass extensively the tissue where they are expected to accumulate (Szczepanowicz et al., 2016). PEGylation of the outer layer of the LbL assembled NDDS is the common method to achieve prolonged circulation. For instance, poly-L-glutamic acid (PGA) and poly-l-lysine (PLL) are assembled layer-by-layer on the surface of paclitaxel-loaded nanoparticles, then surface of the LbL assembled nanoparticles are further PEGylated through the adsorption of the pegylated polyelectrolyte (PGA-g-PEG) as the outer layer to prolong the persistence of the nanoparticles in the circulation, as illustrated in Figure 4(a) (Szczepanowicz et al., 2016). Besides, LbL poly(3,4-ethylenedioxythiophene) (PEDOT)/poly(4-styrenesulfonate) (PSS) nanoparticles have been developed and then further assembled with branched polyethylene glycol (PEG), as illustrated in Figure 4(b) (Cheng et al., 2012). The obtained PEDOT/PSS-PEG nanoparticles are highly stable in the physiological environment and exhibit a stealth-like behavior after intravenous injection with a long blood circulation half-life. As a result, an extremely high in vivo tumor uptake of PEDOT/PSS-PEG attributed to the EPR effect can be observed.

### 4.2. Active targeting

Compared with normal cells and tissues, the expression of specific molecules is significantly high which can be subsequently adopted as targeted receptors for active target anticancer drug delivery, such as the folate receptor (FR), epidermal growth factor receptor (EGFR), CD44 glycoprotein and αvβ3 integrin that are highly expressed on the surface of tumor cell membranes, as well as vascular endothelial growth factor (VEGF) and vascular cell adhesion molecula-1 (VCAM-1) that are highly expressed in tumor vasculature system (Zhong et al., 2017; Semkina et al., 2018; Tulchinsky et al., 2019; Corroyer-Dulmont et al., 2020; Li et al., 2020; Soleymani et al., 2020). The outermost layer of the LBL assembled NDDS can be modified to assemble with specific ligand to target certain receptor and therefore achieve efficient active targeted delivery of anticancer therapeutics.

#### 4.2.1. Folate receptor targeting

**Figure 5.**
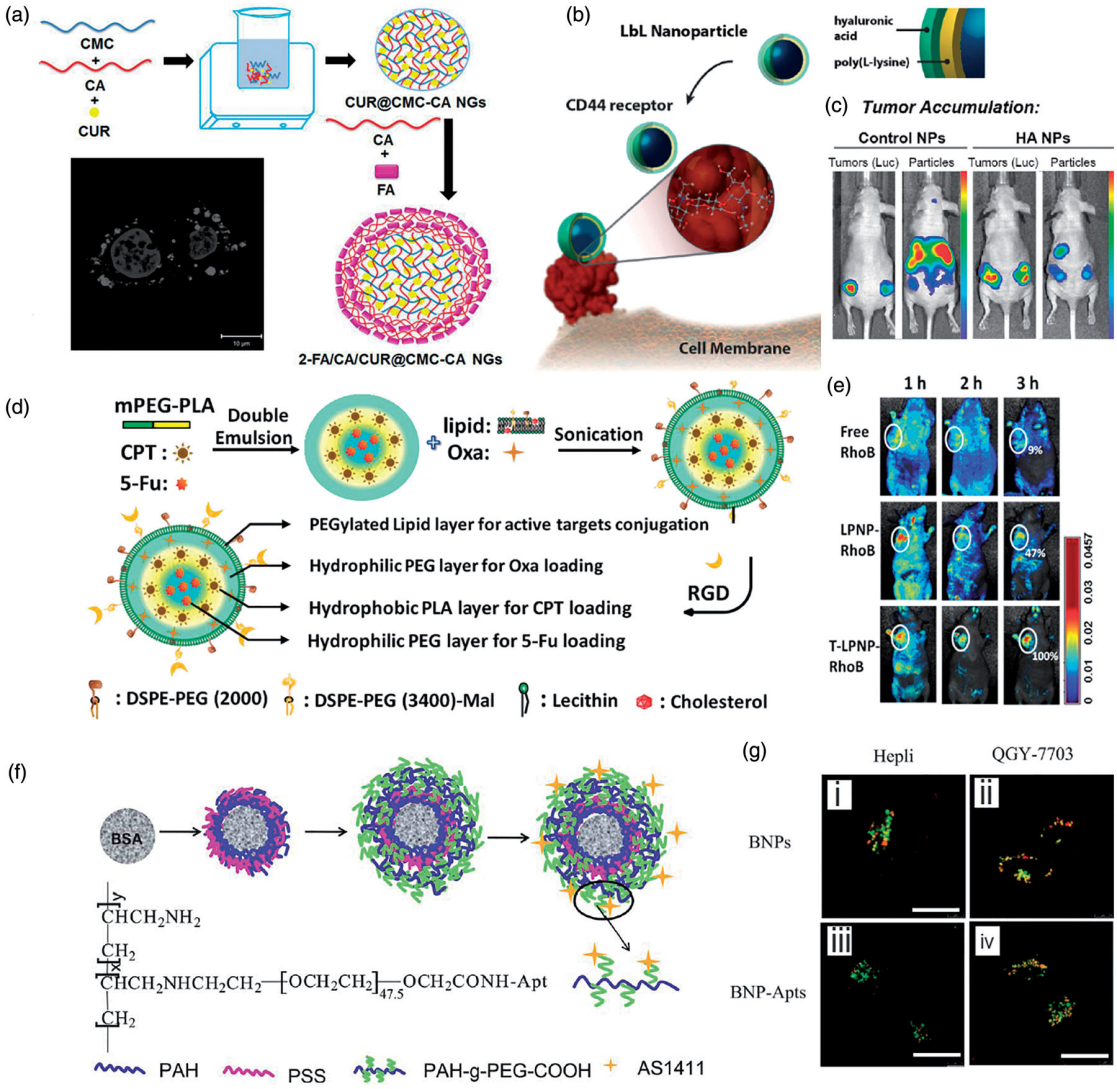
Active targeting of LbL assembled NDDS. (a) Schematic illustration of the LbL assembly of the folate receptor targeted curcumin-loaded folic acid/casein coated carboxymethyl cellulose-casein NGs. (b) Schematics of the LbL assembly of the CD44 receptor targeted hyaluronan/PLL nanoparticles. (c) Whole-animal bioluminescence/fluorescence imaging of tumor co-localization from hyaluronan/PLL nanoparticles compared with dextran sulfate-conjugated control nanoparticles. (d) Schematics of the assembly of RGD targeted RGD-NPs-FOLFIRINOX. (e) Whole body dorsal fluorescence images of nu/nu mice bearing subcutaneous tumors at different time points after the administration by tail vein injection. (f) Schematic illustration of aptamer targeted BSA nanoparticles coated with PAH/PSS multilayers and coupled with aptamer AS1411. (g) CLSM images of Hepli and QGY-7703 cells demonstrating the targeted capacity of assembled AS1411-BSA NPs. (a) Reproduced with permission (Priya et al., 2020). Copyright 2020, Elsevier. (b, c) Reproduced with permission (Dreaden et al., 2014). Copyright 2015, American Chemical Society. (d, e) Reproduced with permission (Li et al., 2015). Copyright 2015, WILEY-VCH. (f, g) Reproduced with permission (Xie et al., 2012). Copyright 2018, Royal Society of Chemistry.

FR is highly expressed on the surface of epithelial tumor cells including ovarian, skin and breast cancer and is one of the common molecular targets (Zhou et al., 2010a; 2010b). For instance, carboxymethyl cellulose (CMC) and casein (CA) nanogels (NGs) encapsulated with curcumin (CUR) have been developed, and further assembled with folic acid (FA) and casein using layer-by-layer (LbL) technique to obtain FA/CA/CUR@CMC-CA NGs for the targeted delivery of curcumin for skin cancer, as illustrated in Figure 5(a) (Priya et al., 2020). Results demonstrate that by assembled with FA, the cellular uptake of NGs can be improved, resulting in enhanced cytotoxicity and apoptotic against MEL-39 melanoma cancer cells overexpressing folate receptors.

#### 4.2.2. CD44 targeting

The high expression of CD44 in tumor cells enables it as a well-characterized targeted receptor for breast and ovarian cancer stem cells, and CD44 can form complexes with hyaluronan (HA) which can trigger the endocytosis by tumor cells (Chen et al., 2017; Gautam et al., 2020). For instance, poly(L-lysine) (PLL) and HA can be assembled layer-by-layer on the surface of carboxylate modified polystyrene nanoparticles to form LbL HA/PLL NPs, as shown in Figure 5(b) (Dreaden et al., 2014). Then taking advantage of the complexation between CD44 and HA, the obtained HA/PLL NPs can selectively bound CD44 *in vitro* with good targeting property, and can diminish cancer cell migration in a receptor-selective manner and co-localized with CD44 receptor *in vivo*, as demonstrated by the bioimaging photos in Figure 5(c).

#### 4.2.3. RGD targeting

The short arginine-glycine-aspartate (Arg-Gly-Asp, RGD) peptide is the smallest sequence expressed by many extracellular matrix proteins and cell membranes that can specifically bind to αvβ3 integrin, therefore can be used to facilitate the active target delivery of anticancer drugs (Wang et al., 2016). For example, LbL assembled nanoparticles have been developed to encapsulate FOLFIRINOX for treating advanced pancreatic cancer with long half-life *in vivo* and high serum stability. The subsequent linkage of tumor targeting RGD polypeptide to the hybrid nanoparticles can enhance the tumor targeting capability, as illustrated in Figure 5(d) (Li et al., 2015). Due to their longer circulation time and better tumor targeting ability *in vivo*, the RGD-NPs-FOLFIRINOX exhibit significantly improved antitumor efficacy compared with free drugs and further enhancement compared with NPs-FOLFIRINOX, with almost no side effects on the major organs in the experimental duration, as demonstrated by the bioimaging photos in Figure 5(e).

#### 4.2.4. Nucleic acid aptamer targeting

Aptamer is an *in vitro* screening technology to obtain oligonucleotide fragments from a library of nucleic acid molecules. Nucleic acid aptamer can fold into a unique three-dimensional structure, which can selectively bind to targeting molecules. Mesoporous silica nanoparticles gave been assembled with thrombin aptamer liposomes. The selective recognition of thrombin aptamer can improve the delivery of docetaxel to tumor cells and therefore significantly inhibit tumor cell proliferation (Gao et al., 2011). Besides, LbL PAH/PSS multilayers are first assembled on the surface of bovine serum albumin (BSA) nanoparticles, then further assembled with PAH-g-PEGCOOH monolayer and subsequently grafted with the aptamer AS1411, which can target the overexpressed nucleolin in the cancer cell membrane, as illustrated in Figure 5(f) (Xie et al., 2012). With the targeting properties, the AS1411-BSA NPs loaded with DOX could more effectively induce the death of liver cancer cells than the free drug, while maintaining the same toxicity to liver normal cells, as demonstrated by the confocal microscopic images in Figure 5(g).

## 5. Controlled release of LbL assembled NDDS

LbL assembled NDDS have unique advantages in achieving controlled release of the encapsulated anticancer drugs. By controlling the specific distribute locations of the therapeutics either in the cores or in different layers, the release of the encapsulated therapeutics can be well tuned spatially (Lynn, 2007; Zhang et al., 2018). Moreover, by introducing multiple stimulus response factors into the LbL assembling process, the LbL assembled NDDS can exhibit various intelligent stimuli-response characteristics, and therefore achieve the stimuli-response release of the encapsulated therapeutics. Plenty of works have been done with significant progress achieved, such as the works done by Prof. Rodrigo Fernando Costa Marques et al., which can be applied to help illustrate the controlled release of encapsulated drugs from the LbL assembled NDDS (Bruneau et al., 2019; Lucena et al., 2020; Brandt et al., 2021). Among all the features of LbL layers, permeability plays a vital role in controlling the release kinetics. Current investigations regarding the release kinetics are mostly orientated toward understanding the parameters that affect the permeability of LbL layers in two-dimensional systems. For instance, ionic strength is one of the most important parameters affecting the structure and morphology of LbL layers. High ionic strengths enhance the permeability of LbL layers to molecules, whereas low salt concentrations decrease the permeability (Antipov et al., 2003). Moreover, thickness can also be controlled by varying the ionic strength of the solution and by the number of layers deposited as well (Sabino et al., 2020). At low ionic strengths, the building block polymers can be elongated at the surface, forming a thinner individual layer thickness and thereby a thinner layer, whereas at high ionic strengths they predominantly form loops and consequently a thicker layer (Sydow et al., 2019). Besides, by modifying the pH and the ionization degree of polyelectrolytes, interactions inside the multilayers can be altered and therefore affecting the permeability. Strong polyelectrolytes possess more charge and in turn form stable multilayers. Weak polyelectrolytes, on the other hand, can be treated by means of high temperature or crosslinking in order to arrive at a functional stability. Typically, the drug release rate is dependent on the solubility and the size of the drug, the number of LbL layers and thickness of the layers, as well as on the type of building block polymers used in the LbL assembling process (Hua et al., 2003; Mevlüt et al., 2012). The controlled modification of release kinetics of LbL assembled NDDS can be used effectively in anti-cancer treatment. By adjusting the permeability through parameters such as ionic strength, pH, crosslinking, thickness, nature and molecular mass of polyelectrolytes, many issues associated with drug formulation and release could be addressed.

### 5.1. Spatially controlled release

**Figure 6.**
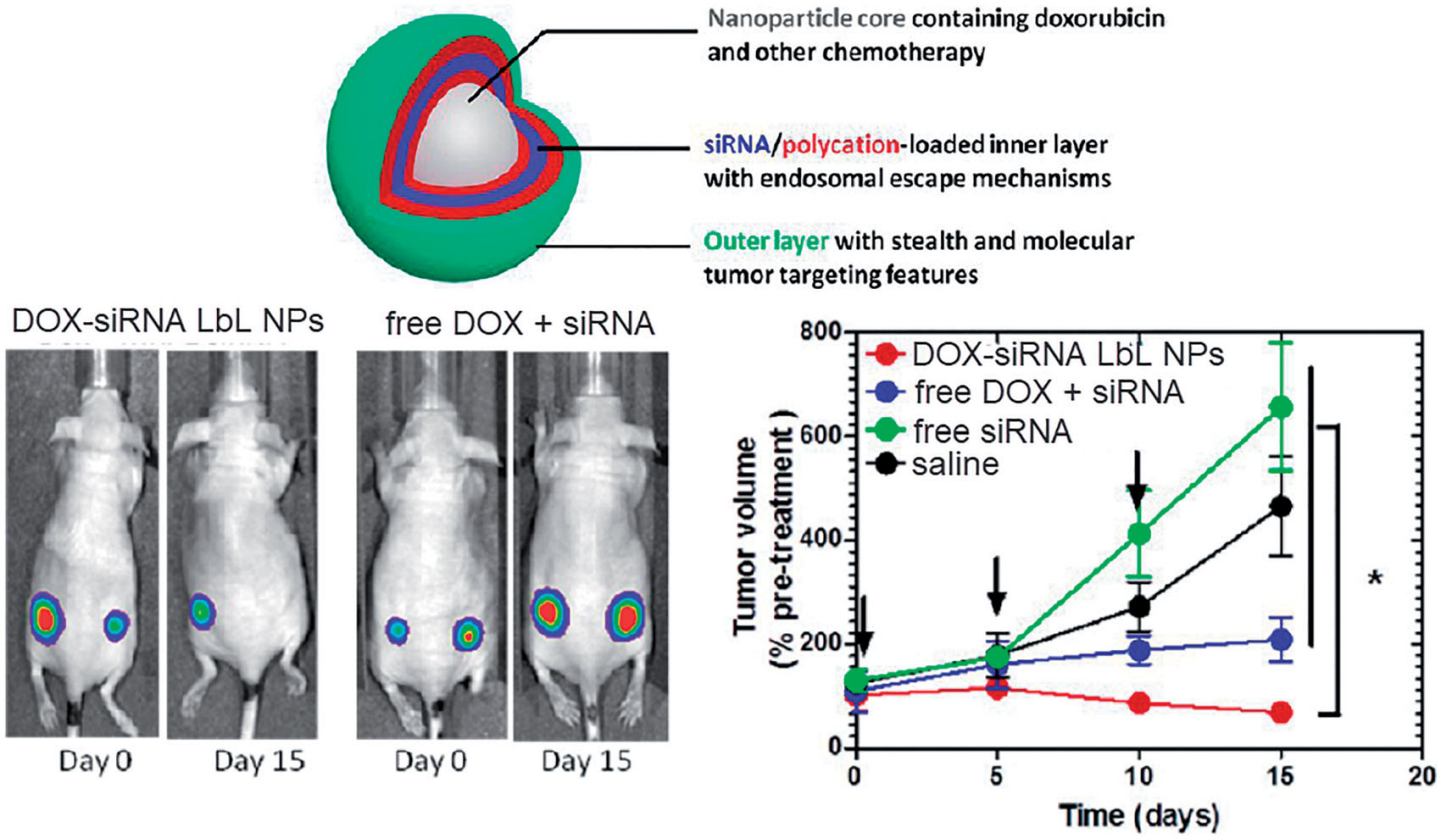
LbL assembled DOX-siRNA co-encapsulated nanoparticles that can achieve the staggered release of DOX and siRNA by controlling the distribute locations of the therapeutics for highly efficient antitumor therapy. Reproduced with permission (Deng et al., 2013). Copyright 2017, American Chemical Society.

The release behavior of LbL assembled NDDS can be regulated by specific controlling the distribute locations of the therapeutics spatially. Specifically, when encapsulating multiple drugs, by regulating the order of the drugs loaded into different layers, the release sequence of different drugs can be effectively controlled (Han et al., 2016). For instance, LbL assembled nanoparticles have been developed to layer-by-layered co-encapsulate siRNA that knocks down a drug resistance pathway in tumor cells and DOX to challenge a highly aggressive form of triple-negative breast cancer. DOX is loaded in the negatively charged phospholipid liposome core of the nanoparticles, while siRNA and poly-L-arginine are alternately depositing on the surface of the nanoparticles to achieve the multilayers, as illustrated in Figure 6 (Deng et al., 2013). Therefore, the release of DOX and siRNA can be staggered, with DOX released 72 hours after the release of siRNA. The efficacy of this DOX-siRNA co-loaded LbL NPs in the treatment of triple negative breast cancer can be 4 times higher than that of free DOX and can significantly reduce the target gene expression in the tumors by almost 80%.

### 5.2. Stimuli-response release

#### 5.2.1. pH responsive release

**Figure 7.**
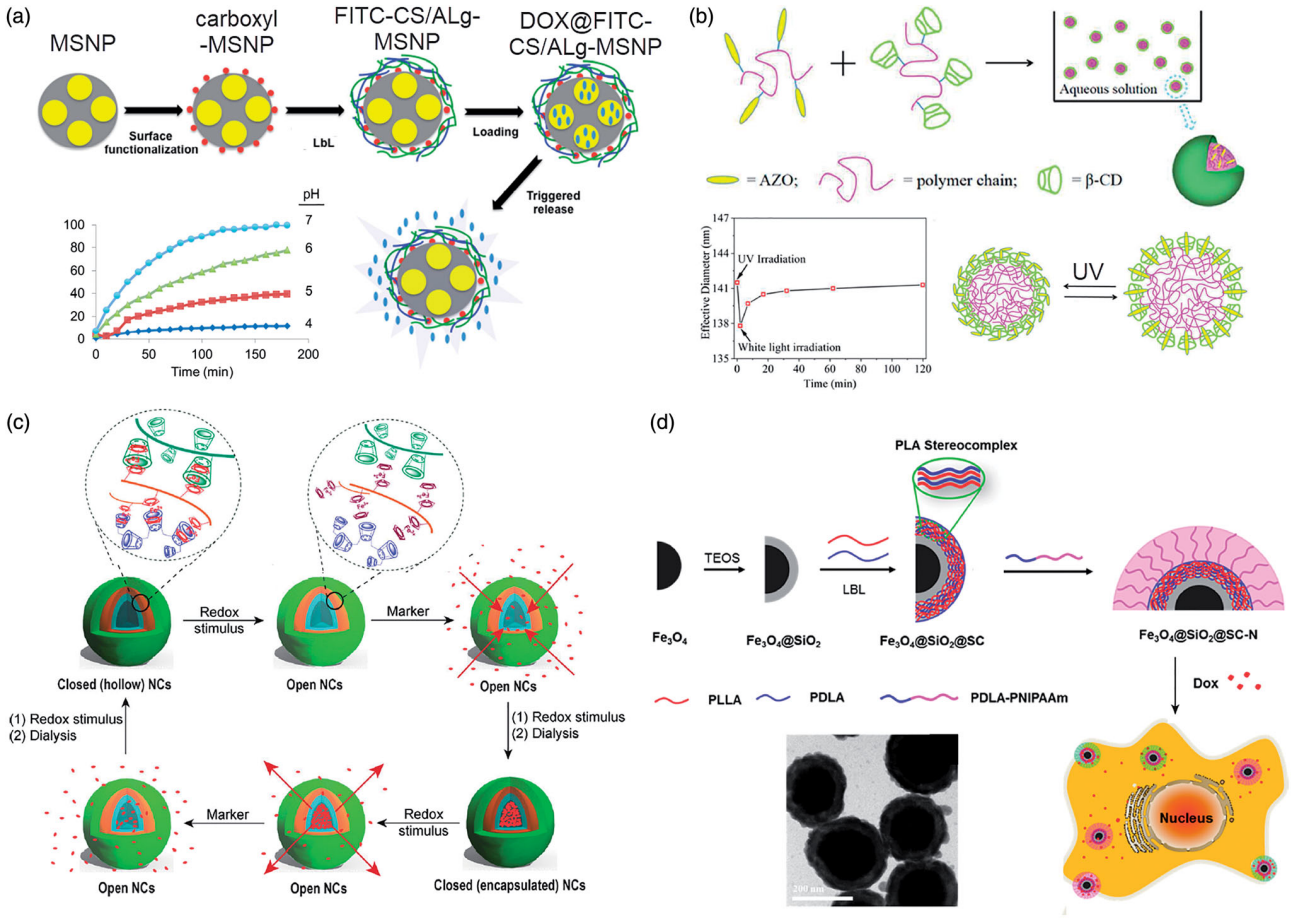
Stimuli-response release of LbL assembled NDDS. (a) LbL assembled FITC-CS/ALg-MSNP with pH responsiveness. (b) LbL assembled AZO/β-CD NPs with light responsiveness. (c) Schematics of the LbL assembled redox reaction responsive NDDS for highly efficient antitumor therapy. (d) LbL assembled PNIPAM/PDLA- Fe_3_O_4_@SiO_2_ nanoparticles for temperature responsive release of DOX. (a) Reproduced with permission (Yilmaz, 2016). Copyright 2016, WILEY-VCH. (b) Reproduced with permission (Pang et al., 2019). Copyright 2019, Frontiers Media S.A. (c) Reproduced with permission (Wajs et al., 2016). Copyright 2016, Springer Nature. (d) Reproduced with permission (Li et al., 2016). Copyright 2019, American Chemical Society.

Due to the special acid environment in tumor cells and tissues, pH responsive release has become a key area in stimuli-response release for LbL assembled NDDS. For NDDS assembled due to the electrostatic interactions between weak polyelectrolytes, varying the pH values can result in changing the degree of ionization and charging of the polyelectrolytes, thereby changing the interaction forces between the positive and negative charges and subsequently change the structure of the LbL nanocompositions. For small molecule drugs that are encapsulated in NDDS by electrostatic interactions, the number of charges that can interact with small molecule drugs will be altered and further lead to pH responsive release of small molecule drugs (Díez-Pascual & Shuttleworth, 2014). Besides, pH-sensitive polymers can also be incorporated in the LbL assembly of NDDS, and changing pH values can cause the disassembly of the multilayered nanocompositions to achieve pH responsive release of anticancer drugs (Du et al., 2015). For instance, by layer-by-layer assemble the pH-sensitive fluorescein isothiocyanate modified chitosan (FITC-CS) and sodium alginate (ALg) on the surface of carboxyl-mesoporous silica nanoparticles (MSNP), LbL assembled FITC-CS/ALg-MSNP with pH responsiveness can be obtained, as illustrated in Figure 7(a) (Yilmaz, 2016). The obtained FITC-CS/ALg-MSNP can increase the drug loading of DOX, and realize the stable loading of the drug at the conventional pH while responsive release of DOX under the low pH values in tumor microenvironment, as shown by the plots in Figure 7(a).

#### 5.2.2. Light responsive release

The intensity and wavelength of light can be adjusted easily and accurately, with the direction, time and irradiation area of the light well controlled externally (Huang et al., 2016). Therefore, the use of photosensitive polymers to assemble LbL NNDS for controlled release anticancer drugs has attracted widespread attention. For example, azobenzene (AZO) with the capacity of reversible isomerization transition has been used as light-responsive materials to LbL assemble with β-cyclodextrin (β-CD) to form nanoparticles, as illustrated in Figure 7(b) (Pang et al., 2019). The developed AZO/β-CD NPs show efficient and effective light responsiveness, including quick responsive time, controllable and gradual recovered process and good fatigue resistance. Moreover, the size of the AZO/β-CD NPs could be adjusted by polymer ratio and light irradiation, which was ascribed to its light-response property. Besides, porphyrin and indocyanine green are used as photosensitive components to prepare LbL assembled NDDS. Under visible light irradiation, the photosensitive components can generate singlet oxygen, which oxidizes the tellurium-containing polymer and transforms it into hydrophilic Te = O groups, resulting in light responsive drug release. And the structure of the LbL assembled NDDS remains intact after the anticancer drug is released, which reduces the side effects of the NDDS *in vivo* during the treatment (Fan et al., 2016).

#### 5.2.3. Redox reaction responsive

Under the action of potential, due to the occurrence of redox reactions can change the potentials and break the charge balance in the LbL assembled nanocompositions, resulting in increasing the numbers of the ions enter the multilayers to increase the osmotic pressure. Consequently, the LbL assembled nanocompositions deforms due to the imbalanced osmotic pressure, which eventually leads to the disintegration and disintegration of the LbL assembled NDDS and trigger the release of the anticancer drugs (Huo et al., 2014). For example, poly(ferrocenylsilannes) (PFS) and poly(acrylic acid) (PAA) can layer-by-layer assembled into hollow nanotubes, while the electro-redox active PFS can help control the release of anticancer drugs through the redox response. After the assembled NDDS reach the tumor tissue, they can achieve pulsed release of drugs under the change of electrical pulses (Song et al., 2013). Furthermore, redox reaction stimuli-responsive hollow nanocapsules (NCs) based on complementary azobenzene/β-CD or Fc/β-CD-grafted dextran polymers have been developed, as illustrated in Figure 7(c) (Wajs et al., 2016). The reversible multipoint crosslinks between the host and guest moieties can be affected by the electro-chemical stimulus and, in turn, altered the macroscopic properties (wall permeability) of the NCs by an on/off switching at the microscopic scale of the inclusion complex, which further facilitate the redox reaction responsive release the encapsulated anticancer drugs.

#### 5.2.4. Temperature responsive release

By incorporating temperature-responsive hydrophilic-hydrophobic phase inversion polymers in LbL assembled NDDS, the encapsulated anticancer drugs can be released under temperature changes. Poly(diallyldimethyl ammonium chloride) (PDAC) and poly(styrene sulfonate) (PSS) have been layer-by-layer assembled on the surface of nanoparticles encapsulated with dexamethasone to obtain temperature-sensitive LbL nanoparticles. The effects of the number of layers, ionic strength, temperature and outermost layer on dexamethasone release have been systematically investigated, demonstrating the feasibility of applying the assembled NDDS as the temperature-responsive platform to release anticancer drugs (Zhou et al., 2014). Besides, temperature-sensitive poly(N-isopropylacrylamide) (PNIPAM) and poly-D-lactic acid (PDLA) have been layer-by-layer assembled on the surface of Fe_3_O_4_@SiO_2_ nanoparticles to form temperature-sensitive LbL NDDS, as illustrated in Figure 7(d) (Li et al., 2016). The release of the encapsulated DOX show temperature-responsiveness and the rates of release can be tuned by variation of external temperatures. The prepared LbL assembled NDDS show great promises on cancer treatment through temperature-responsive controlled-release chemotherapeutics.

## 6. Conclusions and outlooks

The novel LbL assembled NDDS open new perspectives for fabricating high-performance nano platforms for cancer treatment. The resultant NDDS are easy for preparation, capable for size control, and can have high encapsulation efficiency by optimizing the fabrication process. Despite all the attractive advantages, the main drawbacks of the LbL technique are the tedious and time-consuming fabrication process and the waste of materials. Therefore, the development of facile methods with great ease of fabrication and eventually scalable production of LbL assembled NDDS is of both scientific and technical significance. Moreover, LbL assembled NDDS can be used as superior carriers for delivering various anticancer drugs ranging from hydrophilic to lipophilic and are well-suited for controlled and targeted released of the anticancer therapeutics. Besides, LbL assembled NDDS has been applied as a potential strategy for cancer treatment in clinical translation, including for sensitive electrochemical immunoassay (Liu et al., 2012), depressing homeostasis in bone cancer (Hu et al., 2010), photo-chemotherapy (Hashemi et al., 2018), and treating triple-negative breast cancer (Deng et al., 2013). However, at present, most investigations of the LbL assembled NDDS are at pre-clinical stage and far from being applied in clinical trials, which leaves large space and deserves more deep and systematic studies.

There are four major directions for the future developments of LbL assembled NDDS for cancer treatment that we believe worth stating and sharing with researchers: (1) proposing novel techniques to fabricate LbL assembled NDDS; (2) personalized designing of LbL assembled NDDS based on unique characteristics of delivered anticancer drugs; (3) developing methods which can speed up and scale up the LbL fabrication process; (4) speed up the clinical translation. Specifically, more precisely control over the fabrication process needs further explorations to achieve more accurate regulation of the nanostructure and drug distribution of LbL assembled NDDS. For instance, by using microfluidics which can increase the control of the entire fabrication process up to an unprecedented level, it is possible to investigate the sophisticated control over the properties of LbL assembled NDDS (Hamdallah et al., 2020; Ma et al., 2020). Moreover, personalized design of LbL assembled NDDS based on unique characteristics of delivered anticancer drugs is another major direction. For instance, by investigating the special properties of the delivered chemotherapeutic drugs and considering their specific application scenarios in tumor microenvironments, chemical modifications can be used to functionalize LbL assembled NDDS to broaden its applications. For example, near-infrared cyanine dyes can be used to assembled into LbL NDDS modify chitosan. The resultant anticancer drug-loaded LbL assembled NDDS can therefore generate active singlet oxygen under near-infrared irradiation and can be used as novel vehicles for the combination of chemotherapy, photothermal therapy (PTT) and photodynamic therapy (PDT) (Chi et al., 2020; Shen et al., 2020). Besides, aiming at developing methods to speed up and scale up the LbL fabrication process, several techniques directly derived or inspired by the LbL methods are worth considering, such as controlled precipitation (Han et al., 2012), core-mediated in situ PE coacervation (Topbas et al., 2020), polymerization on the surfaces of templates and infiltration and cross-linking on porous templates (Correa et al., 2016; Lyons et al., 2019; Zhang et al., 2019). Moreover, LbL assembled NDDS show great promises in cancer treatment, while its clinical applications are still at its infancy. To speed up the clinical translation, finding more biocompatible materials and developing more biocompatible techniques for the scale-up production of LbL assembled NDDS is worth investigating in the future, since biosafety is one of the top issues when carrying the clinical translation (Gentile et al., 2015).
